# Perceived Neighborhood Environment Impacts on Health Behavior, Multi-Dimensional Health, and Life Satisfaction

**DOI:** 10.3389/fpubh.2022.850923

**Published:** 2022-03-14

**Authors:** Jixiang Liu, Linchuan Yang, Longzhu Xiao, Zhuolin Tao

**Affiliations:** ^1^Department of Urban Planning, School of Architecture and Civil Engineering, Xiamen University, Xiamen, China; ^2^Faculty of Architecture, The University of Hong Kong, Pokfulam, Hong Kong SAR, China; ^3^Department of Urban and Rural Planning, School of Architecture, Southwest Jiaotong University, Chengdu, China; ^4^Department of Architecture and Civil Engineering, City University of Hong Kong, Kowloon, Hong Kong SAR, China; ^5^Faculty of Geographical Science, Beijing Normal University, Beijing, China

**Keywords:** perceived neighborhood environment, health outcome, life satisfaction, health behavior, China, structural equation modeling

## Abstract

The impacts of perceived neighborhood environment on adults' health and life satisfaction have drawn increasing academic attention. However, previous studies usually examine multi-dimensional (physical, mental, and perceived) health and life satisfaction separately, and few studies dealt with them simultaneously. Moreover, limited research revealed the mechanisms behind the effects of perceived neighborhood environment on health and life satisfaction, as well as how such effects are moderated by socio-demographics. Therefore, employing the 2016 China Family Panel Study Dataset and using structural equation modeling, this study delves into the complicated relationships among perceived neighborhood environment, health behavior, health outcomes (i.e., body mass index, self-rated health status, and depression), and life satisfaction. Notably, it considers mediation and moderation simultaneously. It finds: (1) Better perceived neighborhood environment significantly promotes physical activity and reduces sedentary behavior, smoking, and drinking; (2) Health behavior fully mediates the effects of perceived neighborhood environment on health; (3) Perceived neighborhood environment significantly affects life satisfaction both directly and indirectly (through health behavior and health outcomes); (4) Socio-demographics moderate the above relationships. This study disentangles the complicated impacts of perceived neighborhood environment on adults' multi-dimensional health and life satisfaction, thus providing policy makers and practitioners with nuanced knowledge for intervention.

## Introduction

The strikingly rapid urbanization and economic development in China in the recent decades have incurred both benefits and threats to people's health and life satisfaction. From 1978 to 2021, the urbanization rate of China rose from 17.92% (1) to more than 64% (2); China has also been the second-largest economy globally since 2010. As a result, the income, health resources, and quality of life of Chinese urban residents have dramatically improved (3). However, the rapid development has also brought about a variety of problems, e.g., deterioration of environmental quality (4), intensification of socio-economic inequality (5), prevalence of unhealthy lifestyles (e.g., physical inactivity, sedentary behavior, smoking, and excessive drinking) (6), severely challenging people's health and life satisfaction (7, 8). China is currently among the countries with the highest burdens of non-communicable diseases, e.g., cardiovascular disease (9), obesity (10), diabetes (11), cancer (12, 13), and stroke (14). China also encounters an increased prevalence of mental disorder (15). Moreover, unexpectedly, a downtrend in life satisfaction of the general population has been observed along with the unprecedented development in China (7, 16).

As the predominant milieu for urban residents' daily lives, the residential neighborhood has notable health- and life satisfaction-related significance. Neighborhood environment characteristics include physical and social dimensions (17), and they are frequently gauged through objective measures or subjective opinions (perceptions) (18, 19). It remains under dispute if and to what extent perceived neighborhood environment exerts a different effect on people's health or life satisfaction compared with objective neighborhood environment (20). Yet, according to the Theory of Planned Behavior, one's behavior is largely affected by his/her intention, while the intention is decided by attitudes, subjective norms, and perceived behavioral control (21–23). Thus, residents' perceived neighborhood environment is more directly associated with their behaviors and consequently probably affects their health and/or life satisfaction more significantly. Therefore, understanding the impacts of perceived neighborhood environment on health and life satisfaction is indispensable and beneficial for mitigating the abovementioned threats.

Indeed, how the perceived neighborhood environment influences people's multi-dimensional health (e.g., physical, mental, and perceived health) and life satisfaction has drawn increasing academic attention.

As a frequently used indicator for physical health, body mass index (BMI) has been widely confirmed to be impacted by perceived neighborhood environment. For example, Fish et al. found that in Los Angeles, the adults who perceived their residential communities to be unsafe had a significantly higher BMI than their counterparts (24). An international study based on the data from 17 cities across 12 countries, including Australia, USA, UK, Columbia, and China, confirmed that perceived neighborhood safety (safety from traffic or crime) and accessibility to diverse destinations and facilities (e.g., supermarket, school, and transit station) had significant negative effects on adults' BMI (25). Likewise, some other components of perceived neighborhood environment, such as perceived social cohesion (26, 27), neighborhood noise (28), and neighborhood cleanliness (29), were all found to be significantly associated with BMI.

In terms of mental health, perceived neighborhood environment has been examined more frequently as a determinant. Roh et al., for instance, found that perceived neighborhood safety, social cohesion, and overall quality significantly positively affected depressive symptoms of a sample of Korean American adults (30). Zhang et al. also confirmed the significant effects of perceived neighborhood environment on mental health in Shanghai (19). Similarly, Robinette et al. found that perceived neighborhood cohesion was associated with lower level of depression and revealed that it effectively mitigated the detrimental impacts of COVID-19 lockdown on mental health (31).

Self-rated health status represents people's confidence in his/her health condition, which is derived from his/her health literacy, access to health facilities and services, and peer comparisons. Self-rated health has been widely recognized as an effective indicator of health (32). The effects of perceived neighborhood environment on self-rated health have also been examined. For instance, Stronegger et al. found that better-perceived neighborhood environments (e.g., safety, quietness, social cohesion, and better infrastructures) were positively related to higher self-rated health (33). Liu et al. found in Mainland China, Japan, and South Korea that perceived neighborhood facilities, noise, safety, and social cohesion had significant positive effects on self-rated health (34).

Life satisfaction is a broad indicator of well-being and quality of life (35), and it covers multiple domains, such as residential satisfaction, job satisfaction, travel satisfaction, and health-related satisfaction (36). Some studies have explored how perceived neighborhood environment affects life satisfaction. Zhang and Zhang, for instance, found that higher perceived neighborhood environment (e.g., safety, public facilities) were positively associated with life satisfaction (37). Based on their empirical study in Beijing, Ma et al. revealed that higher perceived neighborhood safety, better physical and social environment, and facilities (especially transport-related) significantly positively influenced residents' life satisfaction (38). Perceived neighborhood relationship and social cohesion were found to be significantly positively related to life satisfaction in Rotterdam (39).

Although the above studies provide fruitful insights, three significant research gaps can be identified. First, prior studies have predominantly examined the impacts on multi-dimensional (physical, mental, and perceived) health and life satisfaction separately, and few have dealt with them simultaneously in one study. Given that the perceived neighborhood environment may influence these dimensions of health and life satisfaction differently, a comprehensive understanding of such impacts that integrates the above insights is necessary. Each dimension of health and life satisfaction are vital to people's quality of life. A more comprehensive understanding of how they are influenced by the neighborhood environment can enlighten decision makers with a full-scale knowledge regarding the benefits or detriments of neighborhoods, and thus they can more effectively put forward intervention strategies for promotion of people's quality of life. Second, seldom have researchers explicitly disclosed the mechanisms behind the effects of perceived neighborhood environment on health and life satisfaction, despite some notable exceptions examining the mediating roles of health behavior (predominantly physical activity) on the relationship between perceived neighborhood environment and health outcomes (33, 40, 41). Third, despite the potential population-related heterogeneity, very few studies have investigated whether and how socio-demographics moderate the impacts of perceived neighborhood environment on health and life satisfaction.

Therefore, using the structural equation modeling method and employing the 2016 China Family Panel Study (CFPS) dataset, this study deeply probes into the effects of perceived neighborhood environment on multi-dimensional health and life satisfaction simultaneously, taking both mediation and moderation into consideration. It aims to answer the following three research questions: (1) Does perceived neighborhood environment affect multi-dimensional health and life satisfaction differently? (2) What are the mechanisms behind the effects of perceived neighborhood environment on multi-dimensional health and life satisfaction? (3) Does socio-demographics significantly moderate the abovementioned effects? This study contributes to the research in the following three aspects. First, it delves into multi-dimensional health and life satisfaction simultaneously, thus revealing how the perceived neighborhood environment impacts them differently. Second, through mediation analysis, it shows how perceived neighborhood environment indirectly influences health (through the mediating role of health behavior) and life satisfaction (through the mediating role of both health behavior and health outcomes). Third, through moderation analysis, it uncovers how perceived neighborhood environment affects different population groups (age-, gender-, and hukou-induced) differently.

The remainder of this article unfolds as follows. Section Research Design describes the research design. Section Results presents and analyzes the research results. Section Discussion and Conclusions further discusses the research findings and concludes the article.

## Research Design

### Conceptual Framework and Research Hypotheses

**Figure 1:**
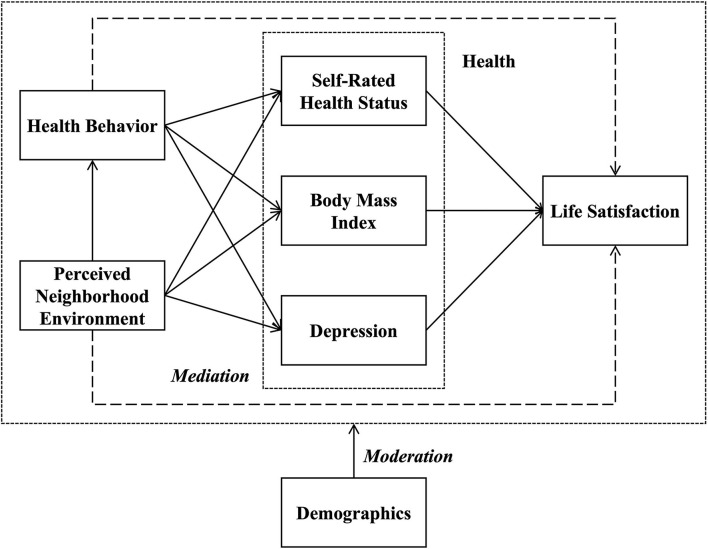
Conceptual framework.

Based on the literature reviewed in the last section, this study proposes the conceptual framework as shown in Figure 1, illustrating the relationships among the perceived neighborhood environment, health behavior, health outcomes (i.e., BMI, self-rated health status, and depression), life satisfaction, and socio-demographics. As presented, perceived neighborhood environment may have direct impacts on the health and life satisfaction of the residents. In the meantime, it probably (1) indirectly impacts health through the mediating effects of health behavior and (2) indirectly impacts life satisfaction through health behavior and health outcomes. Moreover, some socio-demographic features (e.g., age, gender, and urban hukou status) may moderate the abovementioned relationships.

Thus, six hypotheses are proposed based on the framework, which will be verified with structural equation modeling (SEM), as put in Table 1.

**Table 1:** Hypotheses of this study.

**Hypotheses**
**H1**: The perceived neighborhood environment significantly impacts health behavior.
**H2**: The perceived neighborhood environment significantly impacts health outcomes (i.e., BMI, self-rated health status, and depression).
**H3**: The perceived neighborhood environment significantly impacts life satisfaction.
**H4**: The perceived neighborhood environment impacts health outcomes indirectly through the mediating role of health behavior.
**H5**: The perceived neighborhood environment impacts life satisfaction indirectly through the mediating roles of health behavior and health outcomes.
**H6**: Socio-demographics moderate the relationships among perceived neighborhood environment, health behavior, health outcome, and life satisfaction.

### Data Preparation

**Figure 2:**
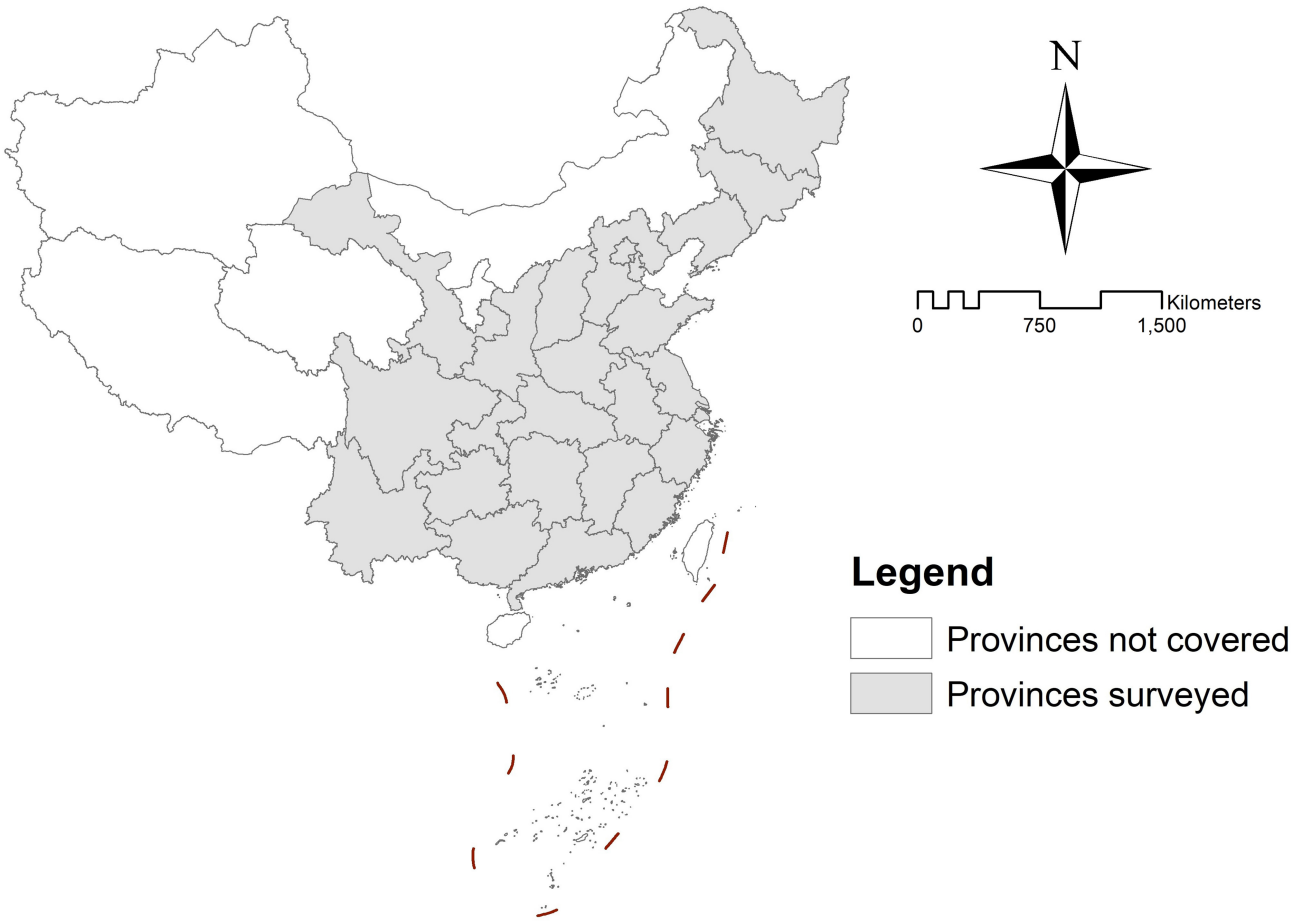
Provinces covered by the CFPS 2016 survey.

The data this study utilizes is extracted from the dataset of China Family Panel Studies (CFPS). The CFPS is a nationally representative longitudinal survey focusing on Chinese communities, families, and individuals, launched by the Institute of Social Science Survey (ISSS) of Peking University in 2010. The CFPS focuses on a wide range of economic and non-economic topics, including economic activities, education outcomes, family dynamics and relationships, migration, and health. By now, there have been 5 waves of surveys conducted in 2010 (baseline survey), 2012, 2014, 2016, and 2018, respectively. The CFPS utilizes a multi-stage, multilevel, implicitly stratified probability sampling method that is in proportion to population size. The surveys cover 25 out of 31 provincial administrative units of China and 95% of the whole population (excluding the residents in Hong Kong, Macau, and Taiwan), which obtain rather representative samples (see Figure 2). The CFPS is one of the largest national longitudinal surveys in China, which provides a valuable database for exploring Chinese people's health and quality of life at the national level. The surveys involve residents in both urban communities and rural villages, and the respondents include children, adolescents, and adults. This study extracted the subsample of adult urban respondents (i.e., those who are 18 years old and above and residing in urban communities) from the CFPS 2016 dataset for analysis. The CFPS 2016, rather than the latter dataset, i.e., CFPS 2018, is chosen because it is the latest dataset with complete perceived neighborhood environment variables available. Then, 21 variables of the CFPS 2016 survey are selected as our key variables, indicating the perceived neighborhood environment, socio-demographic characteristics, health behaviors, health outcomes, and life satisfaction of the respondents. Afterward, the extracted data is cleansed by excluding the responses with outliers or missing values in terms of the selected key variables, thus obtaining a dataset of 5,259 adult respondents for further modeling. The statistical description of each selected variable (mean value and standard deviation (SD) for the continuous variables and the proportion for the categorical variables) can be seen in Table 2.

**Table 2:** Summary statistics of all the variables.

**Latent variables**	**Observed variables**		**Proportion/Mean (SD)**
	**Codes (loadings)**	**Measurement description**	
**Endogenous/mediator variables**			
Life satisfaction	Sa1 (0.871^***^)	What is your perceived social status? (Very low-1-2-3-4-5-Very high)	3.78 (0.98)
	Sa2 (0.922^***^)	How are you satisfied with your life? (Very unsatisfied-1-2-3-4-5-Very satisfied)	3.90 (0.95)
	Sa3 (0.576^***^)	How confident are you about your future? (Not confident at all-1-2-3-4-5-Very confident)	3.97 (0.98)
Depression	De1 (0.709^***^)	How often during the last week did you feel down? (Almost never-1-2-3-4-Almost daily)	1.42 (0.60)
	De2 (0.727^***^)	How often during the last week did you feel that everything seems laborious? (Almost never-1-2-3-4-Almost daily)	1.30 (0.56)
	De3 (0.765^***^)	How often during the last week did you not sleep well? (Almost never-1-2-3-4-Almost daily)	1.21 (0.52)
	De4 (0.748^***^)	How often during the last week did you feel lonely? (Almost never-1-2-3-4-Almost daily)	1.07 (0.48)
	De5 (0.728^***^)	How often during the last week did you feel sad and grieving? (Almost never-1-2-3-4-Almost daily)	1.21 (0.53)
	De6 (0.721^***^)	How often during the last week did you feel that life is unbearable? (Almost never-1-2-3-4-Almost daily)	1.05 (0.45)
Body mass index	BMI	Body mass index (kg/m^2^)	23.11 (3.40)
Self-rated health status	HS	Self-rated health status (Poor-1-2-3-4-5-Excellent)	2.99 (1.13)
Health behavior	HB1 (0.114^***^)	How long did you participate in physical exercise in the past week? (Hours)	2.98 (4.03)
	HB2 (−0.102^***^)	Did you smoke in the past month? (%Yes)	29.21
	HB3 (−0.028^***^)	How long do you usually spend watching TV, movies, or videos every week? (Hours)	13.27 (11.38)
	HB4 (−0.129^***^)	Did you drink more than three times per week in the past month? (%Yes)	10.89
	HB5 (0.276^***^)	How long do you take for housework every day? (Hours)	1.73 (1.64)
**Exogenous variables**			
Perceived neighborhood environment	PBE1 (0.729^***^)	Overall quality of the public facilities (education, medicine, and transportation) surrounding the community (Very bad-1-2-3-4-5-Very good)	3.08 (0.56)
	PBE2 (0.860^***^)	Overall quality of the environment (noise, trash) surrounding the community (Very bad-1-2-3-4-5-Very good)	3.81 (0.61)
	PBE3 (0.725^***^)	Safety surrounding the community (Very bad-1-2-3-4-5-Very good)	3.23 (0.57)
	PBE4 (0.730^***^)	Relationship among the neighbors (Very bad-1-2-3-4-5-Very good)	3.69 (0.63)
	PBE5 (0.807^***^)	Will your neighbors offer help if you need some? (Certainly no-1-2-3-4-5-Certainly yes)	3.54 (0.65)
	PBE6 (0.660^***^)	Emotional attachment to the community (Not at all-1-2-3-4-5-Very much)	3.42 (0.69)
**Moderator variables**			
Socio-demographics	Age (0.207^***^)	Age in years	47.27 (16.64)
	Gender (0.050^***^)	Male = 1, female = 0 (%male)	52.7
	Hu (0.110^***^)	Urban hukou = 1, rural hukou = 0 (%urban hukou)	75.8

*** *p-value < 0.01;*

** p-value < 0.05; * p-value < 0.10.

### Operationalization and Measurement of key Concepts

The key concepts of this study (i.e., perceived neighborhood environment, health behavior, health outcome, life satisfaction, and socio-demographics) are all very broad and contain several latent dimensions. Hence, they are operationalized and measured as latent variables, indicated by the observed variables extracted from the abovementioned dataset, as presented in Table 2. Specifically, life satisfaction and three types of health outcomes, i.e., BMI, self-rated health status, and depression, are selected to act as the endogenous variable in our analysis. While BMI and self-rated health status are observable variables, depression and life satisfaction are both latent variables, which are indicated by 6 and 3 observed variables, respectively. Among them, BMI can indicate people's physical health, self-rated health status can represent people's perceived health or health consciousness, and depression can reflect people's mental health. Meantime, life satisfaction, as a multi-domain indicator, can effectively represent people's overall well-being and quality of life. It is worth noting that to reveal the impacts of perceived neighborhood environment and health behavior on different aspects of residents' health more specifically, the key concept health outcome will not enter the later SEM model directly, while the three sublevel variables will instead. Health outcomes have dual roles in our analyses. They are of key interest to us and will act as endogenous variables. Also, they will act as mediators for the relationship between perceived neighborhood environment and life satisfaction. Health behavior is indicated by 5 observed variables, taking physical activity duration, sedentary behavior duration, smoking, and drinking into account. Health behavior will act as a major mediator in the later analysis. Perceived neighborhood environment, which will act as the exogenous variable, is indicated by 6 observed variables, reflecting the public facilities, safety, and social cohesion of the neighborhoods. All the observed variables of the perceived neighborhood environment are assessed and rated by the respondents, thereby indicating their subjective perceptions of their residing communities. Socio-demographic variables include age, gender, and hukou (household registration system in China) status; they will act as moderators in the further analysis.

To validate the operationalization and measurement, a confirmative factor analysis (CFA) is estimated to test if the latent variables can be adequately explained by their observed variables, with Amos 26. The estimated factor loadings of latent variables on the observed variables are shown in Table 2 (in the brackets after the codes, all with *p* < 0.01).

### Structural Equation Modeling (SEM)

The SEM nowadays is widely applied in such research domains as urban studies and public health. It is capable of incorporating unobservable latent variables and observable indicators and dealing with multiple causes and outcomes, that is, dealing with not only the relationship between independent and dependent variables but also the relationship between dependent variables themselves. Thus, the SEM enables us to identify both the direct and indirect relationships among different variables. Furthermore, the SEM examines the complicated effects simultaneously, thus avoiding the potential biases induced by traditional stepwise regression analyses.

Hence, after operationalizing and validating the key concepts, SEM models are conducted with maximum likelihood estimation in Amos 26. The perceived neighborhood environment acts as the exogenous variables, three selected types of health outcomes and life satisfaction act as endogenous variables. Health behavior acts as the mediator, and socio-demographics as moderators.

The SEM is fitted in two stages. The first stage focuses on the direct and/or indirect impacts of perceived neighborhood environment on the residents' health outcomes and life satisfaction and the mediating role of health behavior. The fitted model in this stage is named the default model.

In the second stage, three socio-demographic variables (age, gender, and hukou status) are entered, and their moderating effects on the aforementioned relationships are examined. Age and gender are fundamental demographical characteristics of people, and their moderating effects are widely documented in health research (42, 43). Meanwhile, Hukou, i.e., the household registration system in China, takes a discriminative attitude toward the internal migrants (without local urban hukou) and excludes them from a variety of social welfares (44). It, therefore, creates a migrant-local dualistic structure in Chinese society. Thus, its potential moderating effects on the built environment-health/life satisfaction associations are also worth exploring. Since lots of categorical variables are involved in the model, this study applies multi-group analysis to examine the moderating effects of these demographics. Specifically, we separately compare the above-mentioned relationships of male vs. female, older adults (45 years old and above) vs. younger adults (44 years old and below), and internal migrants (those without a local urban hukou) and local residents (those with a local urban hukou).

We select several goodness-of-fit measures to assess the performance of the estimated model, including Goodness-of-fit index (GFI), adjusted GFI (AGFI), comparative fit index (CFI), parsimony-adjusted normed fit index (PNFI), parsimony-adjusted comparative fit index (PCFI), root mean square error of approximation (RMSEA), and standardized root mean square residual (SRMR). For a good model, RMSEA should be below 0.08, SRMR should be below 0.05, GFI, AGFI, and CFI should be above 0.90, and PNFI and PCFI should be above 0.50.

## Results

This section presents the model testing and the detailed estimation of the models.

### Model Testing

The default model fits quite well to the data, as indicated by the model fit indices presented in Table 3. Specifically, the RMSEA is 0.061, well below the suggested value 0.08. The SRMR is 0.043, below the suggested value 0.05. In the meantime, the GFI, AGFI, and CFI are all above 0.9, and PNFI and PCFI are both above 0.5. Furthermore, the *p*-value is above 0.05 as suggested. All the indices imply a good model fit. No *post-hoc* modification was conducted due to the good fit of the model.

**Table 3:** Model fit of the default SEM model.

	**P**	**GFI**	**AGFI**	**CFI**	**RMSEA**	**SRMR**	**PNFI**	**PCFI**
**Suggested value**	>0.05	>0.9	>0.9	>0.9	<0.08	<0.05	>0.5	>0.5
**Model value**	0.052	0.929	0.909	0.906	0.061	0.043	0.776	0.780

**Figure 3:**
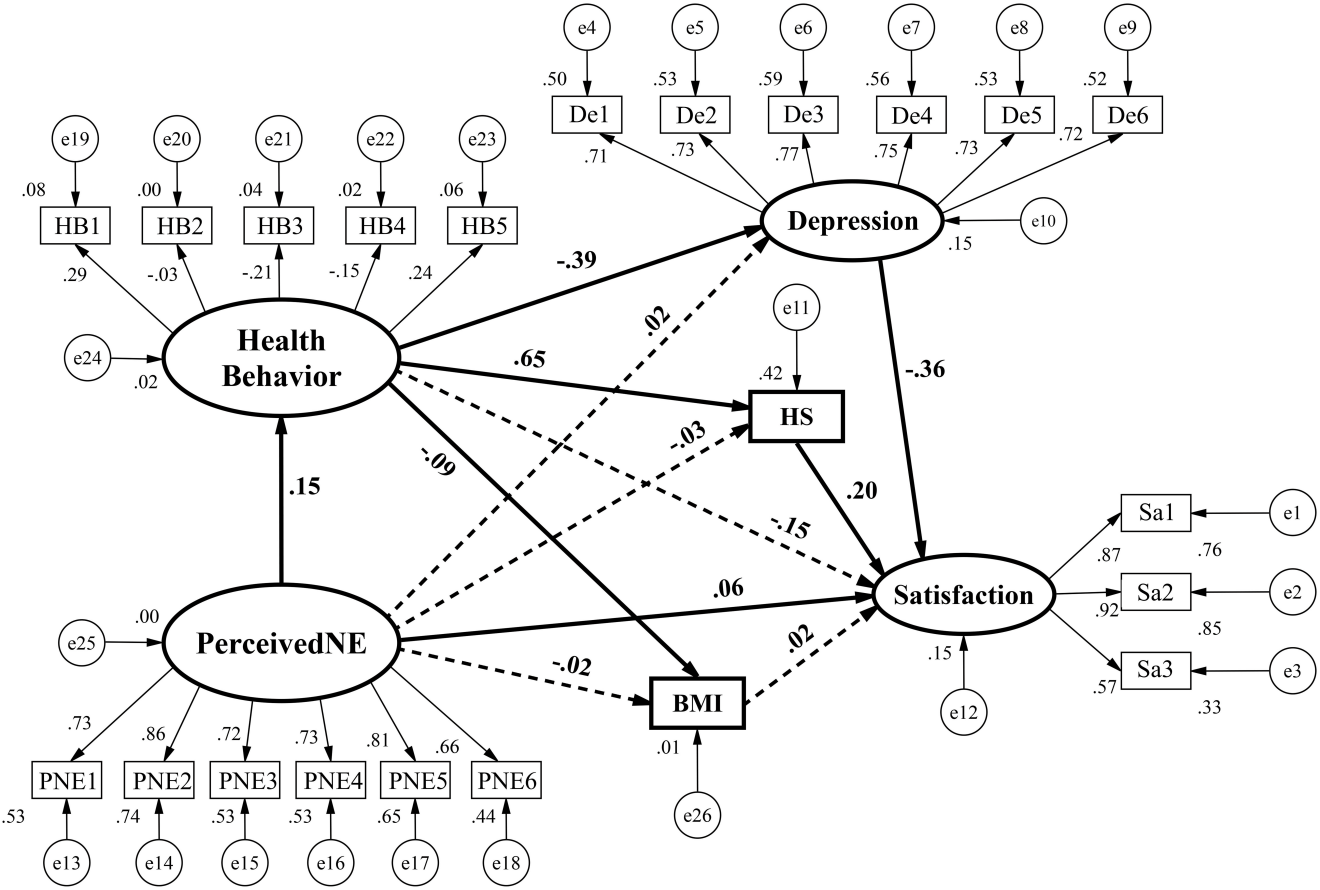
Path diagram of the default model (Full lines indicate weights that are statistically significant at 5% level; otherwise, dash lines).

Our hypothesized model is presented graphically in Figure 3, in which all the standardized regression weights are presented. The ellipses represent the unobservable latent variables, while the rectangles represent the observed variables. For distinguishing the components more clearly, the measurement components are presented by utilizing thin lines while the structural components by bolder lines. Further, the weights that are statistically significant at the 5% level are shown by using full lines, while the insignificant ones are by lines of dashes. As follows, we will present and discuss the complicated relationships among perceived neighborhood environment, health behavior, health outcomes (i.e., BMI, self-rated health status, and depression), and life satisfaction.

### Mediation: Relationships Among Perceived Neighborhood Environment, Health Behavior, Health Outcomes, and Life Satisfaction

As presented (Figure 3 and Table 4), the perceived neighborhood environment has significantly positive effects on health behavior. This finding indicates that the residents who perceive their communities to be with better environment (specifically, better and/or more adequate public facilities, fewer noises or trashes, higher safety, higher social cohesion) tend to have a healthier lifestyle, including more physical activities, less sedentary behavior, and less smoking and/or drinking. This finding is understandable. Such higher-perceived-quality communities can provide adequate, safer, and comfortable open space for the residents, thus attracting more physical activities and simultaneously decreasing their sedentary duration. Moreover, people residing in those higher-perceived-quality communities are usually in higher socio-economic status, and they are widely confirmed to be less likely to consume tobacco or alcohol (45, 46). This finding are consistent with some prior studies (20, 33, 47–50).

**Table 4:** Indirect, direct, and total effects (standardized weights).

**Effects**	**Exogenous/mediator**	**Endogenous/mediator**				
		HB	BMI	HS	Depression	LS
*Indirect*	PNE	–	−0.013^***^	0.098^***^	−0.059^***^	0.002
	HB	–	–	–	–	0.264^***^
	HS	–	–	–	–	–
*Direct*	PNE	0.150^***^	−0.024	−0.032	0.024	0.062^***^
	HB	–	−0.087^***^	0.651^***^	−0.390^***^	−0.150
	BMI	–	–	–	–	0.024
	HS	–	–	–	–	0.195^***^
	Depression	–	–	–	–	−0.355^***^
*Total*	PNE	0.150^***^	−0.037^***^	0.066^***^	−0.035^**^	0.064^***^
	HB	–	−0.087^***^	0.651^***^	−0.390^***^	0.113^**^
	BMI	–	–	–	–	0.024
	HS	–	–	–	–	0.195^***^
	Depression	–	–	–	–	−0.355^***^

*** *p-value < 0.01;*

** *p-value < 0.05;*

*p-value < 0.10. PNE, perceived neighborhood environment; HB, health behavior; HS, self-rated health status; LS, life satisfaction.

The perceived neighborhood environment has no significant direct effects on BMI. By contrast, health behavior significantly negatively affects BMI (−0.09, *p* < 0.01), which echoes many prior studies that confirmed the positive effects of sedentary behavior, smoking, and drinking and negative effects of physical activity as well as their joint effects on BMI (51–56) and/or abdominal-type obesity (particularly for smoking) (57). As shown in Table 4, the indirect effects of perceived neighborhood environment on BMI are also significant (−0.013, *p* < 0.01). The above results indicate that the effects of perceived neighborhood environment on BMI are completely mediated by health behavior.

As expected, more frequent health behavior leads to higher self-rated health status (0.65, *p* < 0.01), which is in line with some previous research (58–63). This finding is reasonable as people who have more physical activities and yet take less smoking, drinking, or sedentary behaviors may be more confident in their health conditions. However, the direct effects of perceived neighborhood environment on self-rated health status are statistically insignificant. Apparently, health behavior completely mediates the effects of perceived neighborhood environment on health status (0.098, *p* < 0.01).

Regarding depression, the direct effects of perceived neighborhood environment are insignificant, yet health behavior has significantly negative effects (−0.39, *p* < 0.01). It proves that health behavior is also beneficial for mental health, in accordance with many prior findings (64–69). In addition, the indirect effects of perceived neighborhood environment on depression are significant (−0.059, *p* < 0.01), suggesting that health behavior plays a fully mediating role in the effects of perceived neighborhood environment on depression, echoing some prior findings (41).

In sum, perceived neighborhood environment has no significant direct effects on the three dimensions of health (i.e., physical health, perceived health, and mental health), yet its indirect effects on them are all statistically significant, and so are the direct effects of health behavior. Hence, the effects of perceived neighborhood environment on multi-dimensional health outcomes are completely mediated by health behavior.

The direct effects of perceived neighborhood environment on life satisfaction are significant (0.06, *p* < 0.01). It is understandable that people who perceive their residential neighborhoods to have higher quality tend to be more satisfied with their life, which coincides with our expectation and previous findings (70, 71). However, health behavior has no significant direct effects on life satisfaction. Likewise, the effects of BMI on life satisfaction are insignificant, which differs from some previous studies that found negative effects of BMI on life satisfaction [e.g., (72, 73)]. In China, some people with higher socio-economic status (e.g., those with higher education attainment or engaging in non-manual jobs) tend to have higher BMI (74, 75), which is different from the western context where overweight or obesity is much more prevalent in lower-socioeconomic-status population subgroups (76, 77). Therefore, this fact can probably confound the relationship between BMI and life satisfaction in China's context. Self-rated health status is significantly positively associated with life satisfaction, suggesting that higher self-rated health status can predict higher life satisfaction. This finding is reasonable and in line with prior findings as well (32). As expected, depression significantly negatively affects life satisfaction, indicating that more frequent depression is detrimental to people's life satisfaction. This finding also echoes some prior studies (78, 79).

Hence, two statistically significant paths exist mediating the effects of perceived neighborhood environment on life satisfaction: (1) perceived neighborhood environment → health behavior → self-rated health status → life satisfaction; (2) perceived neighborhood environment → health behavior → depression → life satisfaction. Specifically, residents in higher-perceived-quality neighborhoods tend to have more frequent health behaviors, higher self-rated health status, less frequent depression, and consequently higher life satisfaction.

### Moderation: Multi-Group Analysis

In the second stage, we conducted multi-group analyses to examine the moderating effects of some typical demographical variables (i.e., age, gender, and hukou status) on the above-discussed relationships. Each of these three variables divides the entire dataset into two subsets, and two individual models are estimated based on the subsets. Tables 5–7 present group differences in the coefficients of each structural path using the critical ratio of the differences between parameters, thus showing the moderating effects of age, gender, and hukou status, respectively.

**Table 5:** Results for moderating effects of age.

**Paths**	**Young adults (≤44)**		**Older adults (>44)**		***z*-score**
	**Estimate**	***P-*value**	**Estimate**	***P-*value**	
PNE → HB	0.169	0.009	0.417	0.003	1.601
PNE → BMI	−0.214	0.024	−0.148	0.120	0.493
PNE → HS	−0.002	0.964	−0.070	0.112	−1.077
PNE → Depression	0.133	0.016	0.044	0.069	−1.488
PNE → LS	−0.141	0.114	0.052	0.053	2.072^**^
HB → BMI	−0.749	0.002	−0.182	0.006	−3.7^***^
HB → HS	0.736	0.000	0.303	0.000	−2.082^**^
HB → Depression	−1.008	0.000	−0.154	0.000	2.906^***^
HB → LS	1.143	0.038	0.094	0.032	−1.896^*^
BMI → LS	−0.006	0.302	0.008	0.117	1.798^*^
HS → LS	−0.007	0.812	0.073	0.038	1.763^*^
Depression → LS	0.253	0.364	−0.317	0.000	−2.006^**^

*** *p-value < 0.01;*

** *p-value < 0.05;*

* *p-value < 0.10*.

As shown in Table 5, in terms of the effects of perceived neighborhood environment on health behavior and three types of health outcomes, no significant differences exist between the young adults (< = 44 years old) and older adults (>44 years old). However, as for the other paths, the coefficients for young adults are significantly different from those of older ones. Specifically, perceived neighborhood environment has no significant effects on the life satisfaction of young adults, yet its influences on older adults' life satisfaction are significant (*p* < 0.1). The effects of health behavior on health (BMI, self-rated health status, and depression) and life satisfaction of young adults are significantly larger than those of older ones, indicating that the health and life satisfaction of young adults are more sensitive and responsive to health behavior than those of older adults. In addition, self-rated health status and depression have no significant effects on young adults' life satisfaction, and yet they significantly affect those of older adults. Thus, age moderates the majority of the structural paths.

As displayed in Table 6, the effects of the perceived neighborhood environment on health behavior vary between females and males; specifically, they are significantly larger for females than males. Interestingly, health behavior significantly lowers females' BMI, but it has no significant impact on males. Meanwhile, significant gender-related differences exist in the effects of health behavior on self-rated health status and life satisfaction and the effects of self-rated health status on life satisfaction. These three paths are all significantly larger for males than females. Therefore, gender also has a moderating role, but its effects are marginal compared with those of age.

**Table 6:** Results for moderating effects of gender.

**Paths**	**Female**		**Male**		**z-score**
	**Estimate**	***P*-value**	**Estimate**	***P*-value**	
PNE → HB	0.624	0.000	0.211	0.197	−1.724^*^
PNE → BMI	−0.184	0.043	0.017	0.854	1.560
PNE → HS	0.021	0.552	−0.026	0.672	−0.666
PNE → Depression	0.018	0.380	−0.013	0.567	−1.017
PNE → LS	0.053	0.017	0.076	0.029	0.557
HB → BMI	−0.233	0.000	0.004	0.923	4.617^***^
HB → HS	0.171	0.000	0.376	0.000	2.946^***^
HB → Depression	−0.069	0.000	−0.094	0.000	−1.565
HB → LS	−0.015	0.275	−0.172	0.011	−2.265^**^
BMI → LS	0.006	0.242	0.009	0.086	0.321
HS → LS	0.091	0.000	0.328	0.000	2.55^**^
Depression → LS	−0.457	0.000	−0.484	0.000	−0.481

*** *p-value < 0.01;*

** *p-value < 0.05;*

* *p-value < 0.10*.

As presented in Table 7, urban hukou status also acts as a significant moderator for most of the paths among perceived neighborhood environment, health behavior, health, and life satisfaction. Specifically, the effects of perceived neighborhood environment on health behavior and three types of health outcomes are significantly different between internal migrants and local residents. In addition, significant variations exist in the influences of health behavior on BMI, self-rated health status, and life satisfaction between the two population subgroups divided by urban hukou status.

**Table 7:** Results for moderating effects of urban hukou status.

**Paths**	**Internal migrants**		**Local residents**		***z*-score**
	**Estimate**	***P*-value**	**Estimate**	***P*-value**	
PNE → HB	0.147	0.527	0.724	0.000	2.12^**^
PNE → BMI	−0.260	0.048	0.021	0.778	1.856^*^
PNE → HS	0.158	0.003	0.366	0.000	−4.568^***^
PNE → Depression	−0.033	0.268	0.046	0.018	2.225^**^
PNE → LS	0.074	0.022	0.085	0.002	0.267
HB → BMI	−0.003	0.952	−0.132	0.000	−2.408^**^
HB → HS	0.171	0.000	0.314	0.000	3.185^***^
HB → Depression	−0.084	0.000	−0.099	0.000	−0.865
HB → LS	0.002	0.921	−0.071	0.021	−1.994^**^
BMI → LS	0.007	0.313	0.005	0.227	−0.235
HS → LS	0.098	0.000	0.182	0.000	1.505
Depression → LS	−0.417	0.000	−0.467	0.000	−0.773

*** *p-value < 0.01;*

** *p-value < 0.05;*

* *p-value < 0.10*.

## Discussion and Conclusions

In this article, we examine the complicated relationships among perceived neighborhood environment, health behavior, health outcome, and life satisfaction. Unlike a large number of the existing studies that focused on single dimensions of health or life satisfaction separately, we investigate three dimensions of health (i.e., physical, mental, and perceived health) and life satisfaction simultaneously, thus providing a comprehensive understanding of the impacts of perceived neighborhood environment on these dimensions of health and life satisfaction. Moreover, different from many prior studies, we consider mediation and moderation simultaneously in our structural equation models. By doing so, we disentangle the complex mechanisms behind the impacts of perceived neighborhood environment on health and life satisfaction as well as the population-related heterogeneity therein.

We have obtained the following important findings.

First, perceived neighborhood environment significantly affects health behavior, thus validating Hypothesis 1 (**H1**). Specifically, residents of neighborhoods with higher perceived quality (i.e., better and/or more adequate public facilities, fewer noises or trashes, higher safety and higher social cohesion) tend to have more physical activities, less sedentary behavior, and less smoking and drinking. This finding confirms the theory of planned behavior, according to which one's attitudes, subjective norms, and perceptions may largely determine his/her behaviors (21–23). Meanwhile, this finding underlines the potential roles of the residential neighborhood environment in the promotion of more healthy lifestyles.

Second, perceived neighborhood environment has no significant direct effects on either of the selected three dimensions of health (i.e., BMI, depression, and self-rated health status). Thus, Hypothesis 2 (**H2**) is rejected. However, the indirect effects of perceived neighborhood environment on these three types of health outcomes through health behavior are all significant, indicating that impacts of perceived neighborhood environment on health are completely mediated by health behavior. Hypothesis 4 (**H4**) is validated. Specifically, people who perceive their communities to be safer, with more accessible public facilities, and with higher social cohesion tend to have more healthy lifestyles and consequently have better physical, mental, and perceived health. These findings imply that in some previous studies [e.g., (24–26, 30, 31)] that did not involve mediation analyses, the true (indirect) effects of perceived neighborhood environment on health were beclouded.

Third, perceived neighborhood environment significantly affects life satisfaction both directly and indirectly (through health behavior and health outcomes). Hypothesis 3 and 5 (**H3** and **H5**) are supported. Specifically, people who perceive their residential neighborhoods to be of higher quality tend to have more healthy lifestyles, have better mental and perceived health, and consequently have higher life satisfaction. These findings corroborate the important roles of perceived neighborhood environment on residents' life satisfaction.

Fourth, through multi-group analysis, we reveal that age, gender, and hukou status have significant moderating effects on most of the relationships among perceived neighborhood environment, health behavior, health, and life satisfaction. Hypothesis 6 (**H6**) is verified. This finding echoes some prior studies [e.g., (42, 43)]. It highlights the existence of population-related heterogeneity in the impacts of perceived neighborhood environment on health and life satisfaction. Researchers have paid predominant attention to the spatial and temporal non-stationarity in the relationships between environment, human behavior, and health/life satisfaction (80–82). This finding suggests that population-related non-stationarity also deserves due academic efforts. It also reminds us that policy makers should avoid the “one-size-fits-all” policies and pay particular attention to vulnerable population subgroups (e.g., the elderly and females).

The above findings provide some practical implications. A better-perceived neighborhood environment can, directly and indirectly, promote life satisfaction. Hence, policy makers and urban practitioners can strive to improve both the physical and social neighborhood environment by measures like enhancing the availability and accessibility of public facilities, maintaining quietness and cleanliness, improving the safety or sense of safety of the neighborhoods, and providing infrastructures and creating opportunities for social interactions within the neighborhoods. The perceived neighborhood environment has no significant direct effects on health. Instead, it significantly indirectly impacts health through the mediating roles of health behavior. Therefore, policy makers and urban practitioners are recommended to realize the health-promoting potentials of the neighborhood environment by emphasizing health behavior. To this end, in addition to the abovementioned interventions toward the neighborhood environment, decision-makers can also formulate some policies and regulations to facilitate healthy behaviors (e.g., physical activity) and discourage unhealthy behaviors (e.g., smoking and excessive drinking). Moreover, the revealed significant moderating effects of socio-demographics can enlighten the policymakers with targeted interventions for certain population subgroups. For example, the perceived neighborhood environment has significant effects on the depression of local residents and yet no effects on that of internal migrants. Hence, in neighborhoods with a higher proportion of internal migrants, such as urban villages in some cities (83), interventions toward environment enhancement may not be sufficient for depression mitigation or prevention. Supplementary measures, e.g., psychological guidance, may also be necessary.

Without any doubt, our study has some limitations that warrant future efforts. First, similar to Liu et al. (84) and Yang et al. (85, 86), the cross-sectional nature of the research design of this study decides that the revealed relationships can only be considered as “causal terms based on plausibility”. Future studies can try to design a longitudinal study to obtain stronger causal relationships. Second, for the protection of respondents' privacy, respondents' detailed addresses are unavailable in the CFPS dataset. Thus, it is impossible to objectively measure the neighborhood environment. In the future, researchers can use appropriate datasets to compare the effects of objective and perceived neighborhood environments on health and life satisfaction. Third, to quantitatively measure one of our core variables, i.e., depression, we only choose six variables from the 20-item scale in the original dataset because the remaining 14 variables are less relevant, following Guo (87). Future studies can take advantage of all the variables to build a more comprehensive construct, e.g., overall mental health condition.

## Data Availability Statement

Publicly available datasets were analyzed in this study. This data can be found here: https://opendata.pku.edu.cn/dataverse/CFPS?language=en.

## Author Contributions

JL, LY, and LX contributed to conception and design of the study. JL and LX performed the statistical analysis. JL organized the database and wrote the first draft of the manuscript. LY, LX, and ZT wrote sections of the manuscript. All authors contributed to manuscript revision, read, and approved the submitted version.

## Conflict of Interest

The authors declare that the research was conducted in the absence of any commercial or financial relationships that could be construed as a potential conflict of interest.

## Publisher's Note

All claims expressed in this article are solely those of the authors and do not necessarily represent those of their affiliated organizations, or those of the publisher, the editors and the reviewers. Any product that may be evaluated in this article, or claim that may be made by its manufacturer, is not guaranteed or endorsed by the publisher.
